# Safety Profile of Primary Intragastric Balloon Placement in Class III Obesity: An MBSAQIP Analysis of 4,555 Patients

**DOI:** 10.1007/s11695-026-08575-8

**Published:** 2026-03-15

**Authors:** Sevag Hamamah, Jamil S. Samaan, Nadine Soliman, Stephanie Nguyen, Nitin Srinivasan, Faizi Hai, Kamran Samakar, Kulmeet K. Sandhu, Kenneth Park, Barham K. Abu Dayyeh, Rabindra R. Watson

**Affiliations:** 1 Department of Medicine, Scripps Mercy Hospital, San Diego, USA; 2 Karsh Division of Gastroenterology and Hepatology, Cedars-Sinai Medical Center, Los Angeles, USA; 3 Faculty of Medicine, Cairo University, Giza, Egypt; 4 Division of Upper GI and General Surgery, Keck School of Medicine, University of Southern California, Los Angeles, USA; 5 Department of Gastroenterology, Scripps Mercy Hospital, San Diego, USA; 6 Division of Minimally Invasive & Bariatric Surgery, Cedars-Sinai Medical Center, Los Angeles, USA

**Keywords:** Intragastric balloon, Class III obesity, Short-term outcomes

## Abstract

**Background:**

Intragastric balloon (IGB) therapy is a minimally invasive endoscopic intervention primarily utilized for weight reduction in patients with Class I and II obesity. While less frequently utilized in individuals with Class III obesity (BMI ≥ 40 kg/m²), IGB placement may be considered as a bridge to bariatric surgery or when surgery is contraindicated or declined. However, data regarding short-term safety outcomes in this higher-risk population remain limited.

**Objective:**

This study aims to compare 30-day safety outcomes after IGB placement in patients with BMI ≥ 40 kg/m² versus BMI < 40 kg/m².

**Methods:**

This was a retrospective cohort study of 4,555 patients undergoing primary IGB placement from 2016 to 2023 using the Metabolic and Bariatric Surgery Accreditation and Quality Improvement Program (MBSAQIP) database. Primary outcomes included hospital length of stay and 30-day rates of outpatient intravenous (IV) treatments, emergency department (ED) visits, readmissions, reoperations, interventions, and mortality. Patients were stratified by BMI ≥ 40 kg/m² and BMI < 40 kg/m² for comparative analysis using propensity score-matching (PSM). 962 pairs were identified for PSM.

**Results:**

Patients with BMI ≥ 40 kg/m² were younger on average and more likely to be male and non-Hispanic Black. This group exhibited a higher prevalence of associated medical problems, including diabetes, obstructive sleep apnea, hypertension, renal insufficiency, immunosuppressant use, thromboembolic events, and therapeutic anticoagulation use. After adjustment for baseline differences, patients with BMI ≥ 40 kg/m² were more likely to require a postoperative hospital length of stay longer than one day (*p* = 0.003). No significant differences in 30-day outpatient IV treatments, ED visits, readmissions, reoperations, interventions, or mortality, were observed.

**Conclusion:**

Patients with BMI ≥ 40 kg/m² exhibited a greater burden of associated medical problems and longer hospital length of stay, but otherwise demonstrated comparable short-term safety outcomes to those with BMI < 40 kg/m². These findings suggest comparable 30-day hospital-based safety outcomes after IGB placement in patients with BMI ≥ 40 kg/m² compared with BMI < 40 kg/m².

**Supplementary Information:**

The online version contains supplementary material available at 10.1007/s11695-026-08575-8.

## Key Points

- Patients with BMI ≥ 40 kg/m², despite a higher prevalence of associated medical problems and longer hospital length of stay following the procedure, experienced similar rates of 30-day outpatient intravenous treatments, emergency department visits, readmission, reoperations, and interventions after intragastric balloon placement compared to those with BMI < 40 kg/m².
- The comparable 30-day safety outcomes of intragastric balloon placement in patients with Class III obesity support short-term procedural safety when used selectively.
- These findings suggest that intragastric balloon placement may be considered as a bridge therapy or selective nonoperative option for patients with Class III obesity; however, conclusions are limited to 30-day safety outcomes and do not address long-term efficacy and durability beyond the scope of the MBSAQIP database.

**Supplementary Information:**

The online version contains supplementary material available at 10.1007/s11695-026-08575-8.

## Introduction

Intragastric balloon (IGB) therapy is a minimally invasive endoscopic intervention for weight loss in individuals with obesity, typically considered for patients who are not candidates for surgery or who seek a reversible alternative [1, 2]. By occupying gastric space, the balloon promotes early satiety and reduced caloric intake [3]. Clinical trials have demonstrated its safety and efficacy, with significant weight loss and metabolic improvement among patients with Class I (BMI 30–34.9 kg/m²) and Class II (BMI 35–39.9 kg/m²) obesity [4, 5]. In patients with Class III obesity (BMI ≥ 40 kg/m²), IGB therapy is often used as a bridge to definitive metabolic and bariatric surgery [6]. Notably, IGB therapy demonstrates considerable heterogeneity in weight-loss response, and a substantial portion of patients experience partial or complete weight regain within 6–12 months after balloon removal, underscoring its role as a temporary and non-definitive intervention [7, 8]. Nevertheless, the safety of IGBs in patients with Class III obesity remains less well defined.

Patients with higher BMI, particularly those with Class III obesity often present with a greater burden of obesity-related medical problems such as diabetes, obstructive sleep apnea, and cardiovascular disease, all of which may contribute to increased procedural risk [9]. Higher BMI has been associated with longer anesthesia times, technical difficulties during endoscopic procedures, and higher rates of thromboembolic and respiratory complications [10–12]. Despite the overall favorable safety profile of IGBs, the physiological strain associated with severe obesity raises questions regarding their safety profile in this subgroup. Moreover, data specific to patients with Class III obesity remain limited, as many pivotal IGB trials either excluded these patients or included them in small numbers, thereby limiting the ability to draw definitive conclusions about short-term safety outcomes in this population. Clarifying the safety of IGB placement in this higher risk population has important clinical implications, as demonstrating acceptable short-term risk may inform patient selection, counselling practices and future guideline recommendations regarding the use of temporary endoscopic weight-loss therapy. Further, demonstrating a favorable safety profile in this population may enhance the role of intragastric balloons as a preoperative weight-loss strategy to reduce operative complexity and improve perioperative safety in metabolic and bariatric surgery.

To address this evidence gap, we analyzed data from the Metabolic and Bariatric Surgery Accreditation and Quality Improvement Program (MBSAQIP), a nationally validated registry of perioperative and 30-day postoperative outcomes from accredited bariatric centers in the United States and Canada. We conducted a multicenter analysis of 4,555 patients undergoing IGB placement, stratified by BMI < 40 and BMI ≥ 40 kg/m². The primary objective was to investigate the association of Class III obesity with short-term procedural risk by comparing 30-day healthcare utilization in these cohorts. Propensity score-matching analysis was employed to adjust for baseline differences in associated medical problems and demographic characteristics.

## Methods

### Data Source

Data were obtained from the MBSAQIP database. The registry is North America’s largest bariatric-specific database capturing annual data on patients undergoing metabolic and bariatric surgery from over 900 accredited centers across the United States and Canada. The database is de-identified and HIPAA compliant collecting preoperative, perioperative and 30-day postoperative outcomes data. The database undergoes routine audits to ensure accuracy and quality.

### Study Population

We performed a retrospective cohort study from 2016 to 2023. The variable CPT_UNLISTED_BALLOON was first introduced in 2016, enabling consistent identification of IGB procedures. The study period extends through 2023 which is the most recent year of available MBSAQIP Participant Use Data Files. A total of 4,555 patients who underwent primary IGB placement were identified. Those undergoing IGB placement from 2016 to 2019 were identified using the variable CPT_UNLISTED_BALLOON. From 2020 to 2023, the variable INIT_PROC_DESC was introduced, with a variable output delineating intragastric balloon procedures. Primary cases were identified using variables CPTUNLISTED_REVCONV from 2016 to 2019 and PROCEDURE_TYPE from 2020 to 2023. Primary intragastric balloon procedures based on these variables were included and patients undergoing revision or conversion procedures were excluded. Patients were stratified based on preoperative BMI into two groups: BMI < 40 kg/m² and BMI ≥ 40 kg/m².

### Outcomes

The primary outcomes were hospital length of stay, 30-day outpatient intravenous (IV) treatments, emergency department (ED) visits, hospital readmissions, reoperations, procedural interventions, and all-cause mortality in patients with BMI ≥ 40 kg/m² compared to those with BMI < 40 kg/m². The variable outpatient IV treatment is defined by the MBSAQIP as symptom-driven administration of IV fluids or medications for in the outpatient setting for documented nausea, vomiting, or fluid, electrolyte, or nutritional depletion, excluding ED or inpatient care. Secondary outcomes included reasons for ED visits, readmissions, reoperations, and interventions.

### Preoperative and Perioperative Variables

Demographic variables include age, sex, and ethnicity categorized as non-Hispanic White, non-Hispanic Black, or Hispanic. Preoperative variables include diagnosis of diabetes, insulin-dependence (if diabetic), active smoking within 1-year, immunosuppressant use, chronic obstructive pulmonary disease (COPD), prior pulmonary embolism (PE), prior deep vein thrombosis (DVT), use of therapeutic anticoagulation, presence of an inferior vena cava (IVC) filter, obstructive sleep apnea, gastroesophageal reflux disease (GERD), prior abdominal surgery, hypertension, hyperlipidemia, venous stasis, history of myocardial infarction, prior percutaneous coronary intervention (PCI), prior cardiac surgery, renal insufficiency, dialysis dependence, and functional status. Perioperative variables included procedure duration in minutes.

###  Statistical Analysis

Descriptive statistics were reported as counts and percentages for categorical variables and as means with standard deviation for continuous variables. Bivariate analysis was performed using student’s t-test when comparing continuous variables as well as Pearson’s chi-squared or Fisher’s exact test when comparing categorical variables. Descriptive and bivariate analysis were performed in IBM SPSS Statistics version 29.0.2.0.

Propensity score-matching (1:1 nearest neighbor without replacement) was performed in R version 4.5.0. BMI ≥ 40 kg/m² was coded as a binary variable and defined as the treatment exposure. Propensity scores were estimated using logistic regression incorporating 18 covariates. Covariates selected for matching was based on observed pre-matching imbalance and the inclusion of clinically relevant confounding variables know to influence perioperative risk. Covariate balance was assessed using standardized mean differences (SMD) before and after matching, with all post-matching SMDs < 0.1 indicating adequate balance (Supplementary Table 1). The total SMD score, which reflects combined imbalance across all covariates, decreased from 0.493 before matching to 0.045 after propensity score-matching. A Love Plot was included to visually demonstrate the improvement in covariate balance following propensity score-matching (Supplementary Fig. 1). A p-value less than 0.05 was considered statistically significant for all analyses.

There was no missing data regarding primary outcomes including 30-day outpatient IV treatments, ED visits, readmissions, reoperations interventions, and mortality. However, reasons for ED visits, readmissions and interventions contained missing data. For ED visits, readmissions, reoperations and interventions, all events were included in the analysis regardless of whether a reason was provided. When evaluating secondary outcomes, including distribution of specific causes for healthcare utilization, only encounters with documented indications were analyzed, and data with missing reasons were excluded. Because the mechanism of missingness could not be determined from the dataset, we did not assume data were missing completely at random or missing at random. Inferential analyses (chi-squared tests, Fisher’s exact and independent t-tests) were performed only on primary outcomes, which had no missing data. Secondary outcomes were summarized using descriptive statistics.

## Results

### Preoperative and Perioperative Factors

Among 4,555 patients who underwent IGB, 962 (21.1%) had a BMI ≥ 40 kg/m². Compared to those with BMI < 40 kg/m², the higher-BMI group was younger (44.6 ± 11.6 vs. 46.2 ± 11.4 years, *p* < 0.001) and had a lower proportion of female patients (71.2% vs. 85.3%, *p* < 0.001). There were no significant differences in Hispanic ethnicity or non-Hispanic White race, though a greater proportion of patients with BMI ≥ 40 kg/m² were non-Hispanic Black (13.6% vs. 8.8%, *p* < 0.001) (Table 1).

**Table 1:** Comparison of demographics and preoperative associated medical problems in patients undergoing intragastric balloon placement, stratified by BMI ≥ 40 kg/m² vs. BMI < 40 kg/m²

Demographics	BMI < 40 kg/m² (*n* = 3593)	BMI ≥ 40 kg/m² (*n* = 962)	*p*-value
Age (mean, SD)	46.2 (11.4)	44.6 (11.6)	< 0.001
Female Sex (n, %)	3066 (85.3)	685 (71.2)	< 0.001
Non-Hispanic White (n, %)	2340 (65.1)	599 (62.3)	0.100
Non-Hispanic Black (n, %)	316 (8.8)	131 (13.6)	< 0.001
Hispanic Ethnicity (n, %) *	282 (9.0)	86 (10.2)	0.275
Preoperative Associated Medical Problems	**BMI < 40 kg/m² (*n*** **= 3593)**	**BMI ≥ 40 kg/m² (*n*** **= 962)**	**p-value**
Diabetes (n, %)	275 (7.7)	149 (15.5)	< 0.001
Insulin-Dependent Diabetes (n, %) *	62 (22.5)	44 (29.5)	0.113
Smoker within One Year (n, %)	182 (5.1)	42 (4.4)	0.373
Immunosuppressant Use (n, %)	27 (0.8)	18 (1.9)	< 0.001
Chronic Obstructive Pulmonary Disease (n, %)	6 (0.2)	2 (0.2)	0.679
History of Pulmonary Embolism (n, %)	9 (0.3)	8 (0.8)	0.015
History of Deep Vein Thrombosis (n, %)	8 (0.2)	8 (0.8)	0.010
Therapeutic Anticoagulation Use (n, %)	20 (0.6)	17 (1.8)	< 0.001
Inferior Vena Cava Filter (n, %)	2 (0.1)	0 (0)	0.464
Sleep Apnea (n, %)	294 (8.2)	190 (19.8)	< 0.001
Gastroesophageal Reflux Disease (n, %)	823 (22.9)	246 (25.6)	0.083
Prior Abdominal Surgery (n, %)	176 (4.9)	25 (2.6)	0.002
Hypertension (n, %)	844 (23.5)	318 (33.1)	< 0.001
Hyperlipidemia (n, %)	468 (13.0)	137 (14.2)	0.324
Venous Stasis (n, %)	7 (0.2)	3 (0.3)	0.449
History of Myocardial Infarction (n, %)	16 (0.4)	3 (0.3)	0.780
Prior Percutaneous Coronary Intervention (n, %)	27 (0.8)	9 (0.9)	0.567
Prior Cardiac Surgery (n, %)	18 (0.5)	8 (0.8)	0.227
Renal Insufficiency (n, %)	3 (0.1)	6 (0.6)	0.004
Dialysis (n, %)	2 (0.1)	1 (0.1)	0.509
Independent Functional Status (n, %)	3588 (99.9)	957 (99.5)	0.041
Procedure Length in Minutes (mean, SD)	15.1 (18.1)	15.46 (16.2)	0.573

*BMI* body mass index, *n* sample size, *%* percentage, *SD* standard deviation, *Denotes missing data within the group, less than the listed sample size

There were significant differences in preoperative medical problems between BMI groups. Patients with BMI ≥ 40 kg/m² had higher rates of diabetes (15.5% vs. 7.7%, *p* < 0.001), immunosuppressant use (1.9% vs. 0.8%, *p* < 0.001), history of PE (0.8% vs. 0.3%, *p* = 0.015), history of DVT (0.8% vs. 0.2%, *p* = 0.010), therapeutic anticoagulation use (1.8% vs. 0.6%, *p* < 0.001), sleep apnea (19.8% vs. 8.2%, *p* < 0.001), hypertension (33.1% vs. 23.5%, *p* < 0.001), and renal insufficiency (0.6% vs. 0.1%, *p* = 0.004). Patients with BMI ≥ 40 kg/m² had lower rates of prior abdominal surgery (2.6% vs. 4.9%, *p* = 0.002). Functional independence was slightly lower in the BMI ≥ 40 kg/m² group (99.5% vs. 99.9%, *p* = 0.041). There were no significant differences between groups in smoking status, COPD, hyperlipidemia, GERD, myocardial infarction, prior PCI or cardiac surgery, IVC filter placement or dialysis dependence. Procedure length was comparable between the two groups.

### Postoperative Outcomes in Matched Cohorts

In the propensity-matched cohort (*n* = 962 per group), patients with BMI ≥ 40 kg/m² were more likely to require a postoperative hospital length of stay greater than one day (4.2% vs. 1.9%, *p* = 0.003) compared to patients with BMI < 40 kg/m² **(**Table 2**)**. However, other 30-day outcomes including rates of outpatient IV treatments (5.4% vs. 5.8%, *p =* 0.692), ED visits (3.8% vs. 4.1%, *p =* 0.815), hospital readmissions (2.4% vs. 1.7%, *p =* 0.258), reoperations (0.6% vs. 0.7%, *p =* 0.781) and postoperative interventions (6.6% vs. 5.3%, *p =* 0.211). One death occurred in the BMI ≥ 40 kg/m² group due to abdominal sepsis, with none in the BMI < 40 kg/m² group (0.1% vs. 0.0%, *p* = 1.00). Covariate balance was achieved across all 18 matching variables, with post-matching SMD showing excellent balance (Supplementary Table 1, Supplementary Fig. 1**)**.

**Table 2:** Postoperative 30-day healthcare utilization following intragastric balloon placement in patients with BMI ≥ 40 kg/m² vs. BMI < 40 kg/m² within propensity score matched cohorts

Postoperative 30-day Outcomes	BMI < 40 kg/m² (*n* = 962)	BMI ≥ 40 kg/m² (*n* = 962)	*p*-value
Greater than One Day to Discharge from Procedure (n, %)	18 (1.9)	40 (4.2)	0.003
Outpatient Intravenous Treatment (n, %)	56 (5.8)	52 (5.4)	0.692
Emergency Department Visit (n, %)	39 (4.1)	37 (3.8)	0.815
Readmission (n, %)	16 (1.7)	23 (2.4)	0.258
Reoperation (n, %)	7 (0.7)	6 (0.6)	0.781
Intervention (n, %)	51 (5.3)	64 (6.6)	0.211
Death (n, %)	0 (0)	1 (0.1)	1.000

*BMI* body mass index, *n* sample size, *% *percentage

### Reasons for ED Visits, Readmission, Reoperations, and Interventions

Reason information was frequently unavailable, with only 83 of 197 ED visits (42.1%), 31 of 90 readmissions (34.4%), and 82 of 313 postoperative interventions (26.2%) having a documented reason. The most common causes of ED visits were nausea, vomiting, and dehydration (7/15, 46.7% in patients with BMI ≥ 40 kg/m² vs. 37/68, 54.4% in patients with BMI < 40 kg/m²), followed by abdominal pain (4/15, 26.7% vs. 14/68, 20.6%) (Table 3).

**Table 3:** Reasons for emergency department visits, readmissions, reoperations, and interventions in IGB patients with BMI < 40 kg/m² and BMI ≥ 40 kg/m²

Emergency Department Visit Diagnosis	BMI < 40 kg/m² (*n* = 68)	BMI ≥ 40 kg/m² (*n* = 15)
Nausea, Vomiting, Dehydration (n, %)	37 (54.4)	7 (46.7)
Abdominal Pain (n, %)	14 (20.6)	4 (26.7)
Other (not listed) (n, %)	11 (16.4)	1 (6.6)
Cardiac Cause (n, %)	1 (1.5)	0 (0)
Findings within Normal Limits (n, %)	1 (1.5)	0 (0)
Gastritis (n, %)	0 (0)	1 (6.6)
Musculoskeletal Pain (n, %)	1 (1.5)	2 (13.3)
Oral Intake Intolerance (n, %)	1 (1.5)	0 (0)
Abdominal Sepsis (n, %)	1 (1.5)	0 (0)
Patient Intolerance (n, %)	1 (1.5)	0 (0)
Readmission Diagnosis (*n* = 31) *	**BMI < 40 kg/m² (***n* **= 20)**	**BMI ≥ 40 kg/m² (*n*** **= 11)**
Nausea, Vomiting, Dehydration (n, %)	6 (30.0)	6 (54.5)
Abdominal Pain (n, %)	6 (30.0)	1 (9.1)
Oral Intake Intolerance (n, %)	1 (5.0)	0 (0)
Obstruction (n, %)	0 (0)	1 (9.1)
Other (not listed) (n, %)	3 (15.0)	0 (0)
Patient Intolerance (n, %)	2 (10.0)	2 (18.2)
Gastrointestinal Tract Bleeding (n, %)	0 (0)	1 (9.1)
Cardiac Cause (n, %)	1 (5.0)	0 (0)
Renal Failure (n, %)	1 (5.0)	0 (0)
Reoperation Type (*n* = 56)	**BMI < 40 kg/m² (*n*** **= 50)**	**BMI ≥ 40 kg/m² (*n*** **= 6)**
Other Bariatric Surgery Reoperation (n, %)	39 (78.0)	3 (50.0)
Other-Abdominal Operation (n, %)	9 (18.0)	0 (0)
Takedown or Reversal of Intragastric Balloon (n, %)	2 (4.0)	3 (50.0)
Intervention Type (*n* = 313)	**BMI < 40 kg/m² (*n*** **= 249)**	**BMI ≥ 40 kg/m² (*n*** **= 64)**
Other Intervention (n, %)	90 (36.1)	19 (29.7)
Intragastric Balloon Removal (n, %)	53 (21.3)	13 (20.3)
Intraluminal Therapeutic Procedure (n, %)	64 (25.7)	23 (35.9)
Therapeutic Endoscopy (n, %)	18 (7.2)	5 (7.8)
Diagnostic Endoscopy (n, %)	13 (5.2)	3 (4.7)
Intragastric Balloon Adjustment/Placement (n, %)	10 (4.0)	1 (1.6)
Placement of Percutaneous Drain (n, %)	1 (0.4)	0 (0)
Intervention Reason (*n* = 82) *	**BMI < 40 kg/m² (*n*** **= 67)**	**BMI ≥ 40 kg/m² (*n*** **= 15)**
Patient Intolerance (n, %)	21 (31.3)	7 (46.7)
Nausea, Vomiting, Dehydration (n, %)	17 (25.4)	2 (13.3)
Abdominal Pain (n, %)	8 (11.9)	1 (6.7)
Planned Removal per Protocol (n, %)	6 (9.0)	2 (13.3)
Other (not listed) (n, %)	6 (9.0)	1 (6.7)
Oral Intake Intolerance (n, %)	5 (7.5)	0 (0)
Patient Request (n, %)	2 (3.0)	0 (0)
Inadequate Weight Loss (n, %)	1 (1.5)	0 (0)
Gastroparesis (n, %)	0 (0)	1 (6.7)
Dysphagia (n, %)	0 (0)	1 (6.7)
Abdominal Sepsis (n, %)	1 (1.5)	0 (0)

*BMI* body mass index, *n* sample size, *%* percentage. *Data were limited due to missing values: diagnoses were available for 82 of 197 ED visits, 31 of 90 readmissions, and 82 of 313 interventions. Percentages are reported relative to events with available diagnoses or reasons, not the total study population

Readmissions were most often due to nausea, vomiting, and dehydration (6/11, 54.5% vs. 6/20, 30.0%) and patient intolerance (2/11, 18.2% vs. 2/20, 10.0%). Reoperations were less common, with other bariatric surgeries being the most frequent indication (3/6, 50.0% vs. 39/50, 78.0%). Among postoperative interventions, intraluminal therapeutic procedures (23/64, 35.9% vs. 64/249, 25.7%) and intragastric balloon removal (13/64, 20.3% vs. 53/249, 21.3%) were most common. The most frequent reasons for intervention were patient intolerance (7/15, 46.7% vs. 21/67, 31.3%) and nausea, vomiting, or dehydration (2/15, 13.3% vs. 17/67, 25.4%).

## Discussion

In this large, multicenter MBSAQIP analysis of 4,555 primary IGB placements, Class III obesity was associated with similar 30-day safety outcomes after propensity score-matching. Among matched cohorts (*n* = 962 per group), rates of 30-day outpatient IV treatments (5.4% vs. 5.8%, *p* = 0.692), ED visits (3.8% vs. 4.1%, *p* = 0.815), hospital readmissions (2.4% vs. 1.7%, *p* = 0.258), reoperations (0.6% vs. 0.7%, *p* = 0.781), and procedural interventions (6.6% vs. 5.3%, *p* = 0.211) were comparable. One death occurred in the BMI ≥ 40 kg/m² group and none in the BMI < 40 kg/m² group (0.1% vs. 0.0%, *p* = 1.000). These results suggest that, despite a greater burden of associated medical problems in Class III obesity, short-term safety following IGB placement is comparable across BMI categories. Importantly, comparable short-term safety outcomes do not imply equivalent procedural tolerance, as the full spectrum of intolerance symptoms and elective early balloon removal are incompletely captured within the MBSAQIP database. Despite these comparable findings between cohorts, patients with Class III obesity were more likely to require a postoperative hospital stay exceeding one day (4.2% vs. 1.9%, *p =* 0.003), a finding that has not been well characterized in prior IGB literature.

Current American Society for Gastrointestinal Endoscopy (ASGE) and European Society of Gastrointestinal Endoscopy (ESGE) guidelines recommend IGB placement as a primary endoscopic therapy for select patients with Class I and II obesity, citing modest weight loss and an acceptable short-term safety profile [13]. Most adverse events such as nausea, vomiting, and abdominal discomfort are self-limited, while serious complications, such as balloon deflation, migration, or perforation, are rare. Previous large-scale studies and meta-analyses have primarily included patients with Class I–II obesity, where IGB therapy has demonstrated a favorable safety profile [14]. In a meta-analysis by Imaz et al., serious complication rates were low, with nausea or vomiting (8.6%) and abdominal pain (5%) being the most common adverse events. Early balloon removal occurred in 4.2% of cases, nearly half of which were voluntary [15]. Similarly, a pilot study in patients with lower BMI reported no serious complications, with only one readmission (5.3%) [16]. In a randomized trial assessing safety and efficacy of the ORBERA IGB, Courcoulas et al. reported an 18.8% early device removal rate by 6 months, half of which were patient-initiated [4]. Another post-marketing multicenter study of 258 adults with BMI 30–40 kg/m² treated with the ORBERA IGB had an 8.9% rate of device-related adverse events meeting their primary endpoint with no new risks identified [17]. While supportive of IGB safety in Class I–II obesity, these studies lack direct comparison with Class III obesity.

 Available data on the safety of IGB placement in patients with higher BMI thresholds (BMI > 40 kg/m² and BMI > 50 kg/m²) remain limited to small-scale studies, variable follow-up durations, and absence of direct comparisons between BMI groups [18, 19]. A 6-month randomized crossover trial in 32 patients with Class III obesity reported no serious complications and greater weight loss versus sham diet (p < 0.001) [20]**.** Another retrospective study of 46 patients with BMI > 40 kg/m² examined safety, weight loss, and progression to definitive bariatric surgery [21]. Readmission rates in this cohort was 21.7%, primarily due to nausea, vomiting, and dehydration. Additionally, 15.2% underwent early balloon removal. All readmissions occurred within 52 days post-IGB insertion, with a median number of 8 days post-procedure. Additionally, 6 (13%) patients required placement of a second IGB and 1 (2.2%) received a third IGB. Similarly, a retrospective study of 74 patients with BMI > 50 kg/m² treated with IGB prior to metabolic surgery reported a 21.6% complication rate primarily due to nausea, vomiting and dehydration with a reintervention rate of 5.4% [19]. Lastly, a study of 94 patients with BMI > 50 kg/m² undergoing IGB placement reported adverse events including abdominal pain in 21.3%, vomiting in 17%, and early balloon removal in 11.7% of cases [22]. However, direct comparisons with our findings are limited by several factors, including differences in study design, absence of BMI-stratified safety comparisons, and the nature of our data source.

 Furthermore, these studies do not report hospital length of stay following IGB placement. While not recommended as definitive treatment for patients with Class III obesity, IGBs are increasingly employed as bridge therapy in high-risk surgical candidates [13]. These guidelines support integrating IGBs into individualized, risk-stratified obesity management. While patients with Class III obesity are often perceived to be at higher procedural risk due to associated medical comorbidities and technical challenges, our findings indicate that short-term safety following IGB placement is comparable across the BMI spectrum. However, the observed difference in length of stay suggests that postoperative recovery needs and early hospital-based monitoring may vary by BMI, even when major complications do not. These findings suggest that the safety profile of IGBs may extend to appropriately selected patients with more advanced obesity. Collectively, these results have potential implications for informing patient selection, counseling practices, and future guidelines recommendations while supporting the short-term safety of IGBs as preoperative weight-optimization tools in this higher BMI population as a bridge to surgery or in select nonoperative scenarios.

 This study has several limitations. The retrospective, observational design precludes causal inference and is subject to residual confounding despite propensity score-matching. Patients were not randomized, and clinical decisions regarding ED visits, readmissions, or procedural interventions were left to provider discretion, introducing potential center-level variability. Differences in institutional protocols, documentation practices, and coding accuracy may also affect data consistency. Selection bias remains a concern, as the decision to pursue IGB therapy may have been influenced by unmeasured factors such as patient preference or institutional eligibility criteria. Furthermore, device-specific outcomes could not be evaluated, as the MBSAQIP registry does not distinguish between intragastric balloon types, which differ in volume, dwell time, and design. This device heterogeneity limits granularity in evaluating safety across specific balloon systems and may affect generalizability to specific device types.

Additionally, MBSAQIP captures only 30-day postoperative outcomes, limiting assessment of long-term efficacy, weight loss durability, and medium- to long-term complications. The dataset also contained missing information regarding the indications for ED visits, readmissions, reoperations, and interventions. This constrained our ability to fully characterize the clinical spectrum and true percentages of specific adverse events within the total study population. Importantly, the MBSAQIP does not capture symptom severity and clinical rationale for procedural interventions. As a retrospective analysis of a large national database, this study is constrained by the lack of granular symptom reporting with primarily events resulting in ED visits, readmissions, reoperations, interventions, or death being captured. In conjunction with missing reasons for ED visits, readmissions, and interventions, this limits the ability to fully interpret the degree of intolerance in this higher-risk population. Although outpatient IV treatments for dehydration, nausea, vomiting and electrolyte depletion may serve as a proxy for clinically significant intolerance, this variable reflects only symptom-driven escalation of care and does not capture intolerance symptoms managed more conservatively with oral or transdermal medications. As highlighted by prior smaller scale studies discussed earlier, intolerance symptoms such as persistent nausea, vomiting, abdominal pain, and dehydration leading to early balloon removal can occur with appreciable frequency, particularly among patients with higher BMI. Consequently, the low absolute event rates observed in our study may reflect underrepresentation of clinically significant intolerance and other adverse events that did not result in IV treatments or hospital-based encounters. Similarly, although patients with Class III obesity demonstrated a higher likelihood of hospital length of stay longer than one day, MBSAQIP does not capture the clinical reasons underlying prolonged hospitalization. As a result, we were unable to differentiate between observation for intolerance symptoms, institutional practice variation or precautionary monitoring, limiting the interpretation of the mechanisms driving this finding. Therefore, the observed variance in length of stay should not be construed as indicative of increased procedural complexity or more severe complications.

 Interpretation of the null findings should take into account effect size and the relatively low frequency of 30-day healthcare utilization. The matched cohort size provides reasonable precision for detecting moderate differences between BMI groups. However, given the low baseline incidence of postoperative events, the study may not have been powered to detect small absolute differences. Therefore, while no significant differences were observed between BMI categories, the possibility of undetected minor differences in the uncommon postoperative ED visits, readmissions, reoperations, interventions, and mortality should be acknowledged. Nonetheless, this remains the largest BMI-stratified analysis of IGB outcomes to date, offering greater statistical power to detect short-term safety signals in patients with Class III obesity.

## Conclusion

Class III obesity was associated with longer hospital stay, but no significant difference in 30-day outpatient IV treatments, ED visits, readmissions, reoperations interventions or mortality following IGB placement in this large, multicenter cohort study. These findings suggest that short-term safety outcomes are comparable across BMI categories after adjustment for baseline characteristics and associated preoperative medical problems. These findings reflect short-term safety outcomes in the BMI ≥ 40 kg/m² population and should not be construed as evidence advocating for IGB as definitive treatment for Class III obesity, but rather as an option for bridging therapy to definitive metabolic surgery or carefully selected nonoperative scenarios. Prospective studies, including randomized controlled trials with longer follow-up, are needed to assess the safety, durability, and clinical utility of IGBs more definitively in this patient population.

## Supplementary Information

Below is the link to the electronic supplementary material.

Supplementary File 1 (PDF 75.0 KB)

## Data Availability

Data is available to participating centers upon request from the Metabolic and Bariatric Surgery Accreditation and Quality Improvement Program (MBSAQIP).
