# Supplementary material for: Safety Profile of Primary Intragastric Balloon Placement in Class III Obesity: An MBSAQIP Analysis of 4,555 Patients
Source: Obes Surg. 2026 Mar 15;36(4):1757–65. doi: 10.1007/s11695-026-08575-8 (PMC13083416; doi:10.1007/s11695-026-08575-8)
Supplement: Supplementary file 1 — (PDF 75.0 KB) [file 11695_2026_8575_MOESM1_ESM.pdf]

Supplementary Table 1. Standard Mean Differences After Propensity Score Matching of 18 Matched Covariates.

**COVARIATES**	**STANDARD MEAN DIFFERENCES**
Age Greater Than 45	0.019
Female Sex	0.005
Diabetes Mellitus	< 0.001
Immunosuppressive Use	0.008
Non-Hispanic Black	0.012
History of Pulmonary Embolus	0.069
History of Deep Vein Thrombosis	0.038
Therapeutic Anticoagulation Use	0.043
Sleep Apnea	0.024
Hypertension	0.002
Prior Abdominal Surgery	0.006
Independent Functional Status	0.032
Renal Insufficiency	0.065
Gastroesophageal Reflux Disease	0.012
Prior Percutaneous Coronary Intervention	0.035
History of Myocardial Infarction	0.046
Chronic Obstructive Pulmonary Disease	< 0.001
Smoker within One Year	0.066
**Total Pre-Match Distance**	**Total Post-Match Distance**
0.493	0.045

Supplementary Figure 1. Love Plot Demonstrating Standard Mean Differences Prior to and After Propensity Score Matching.

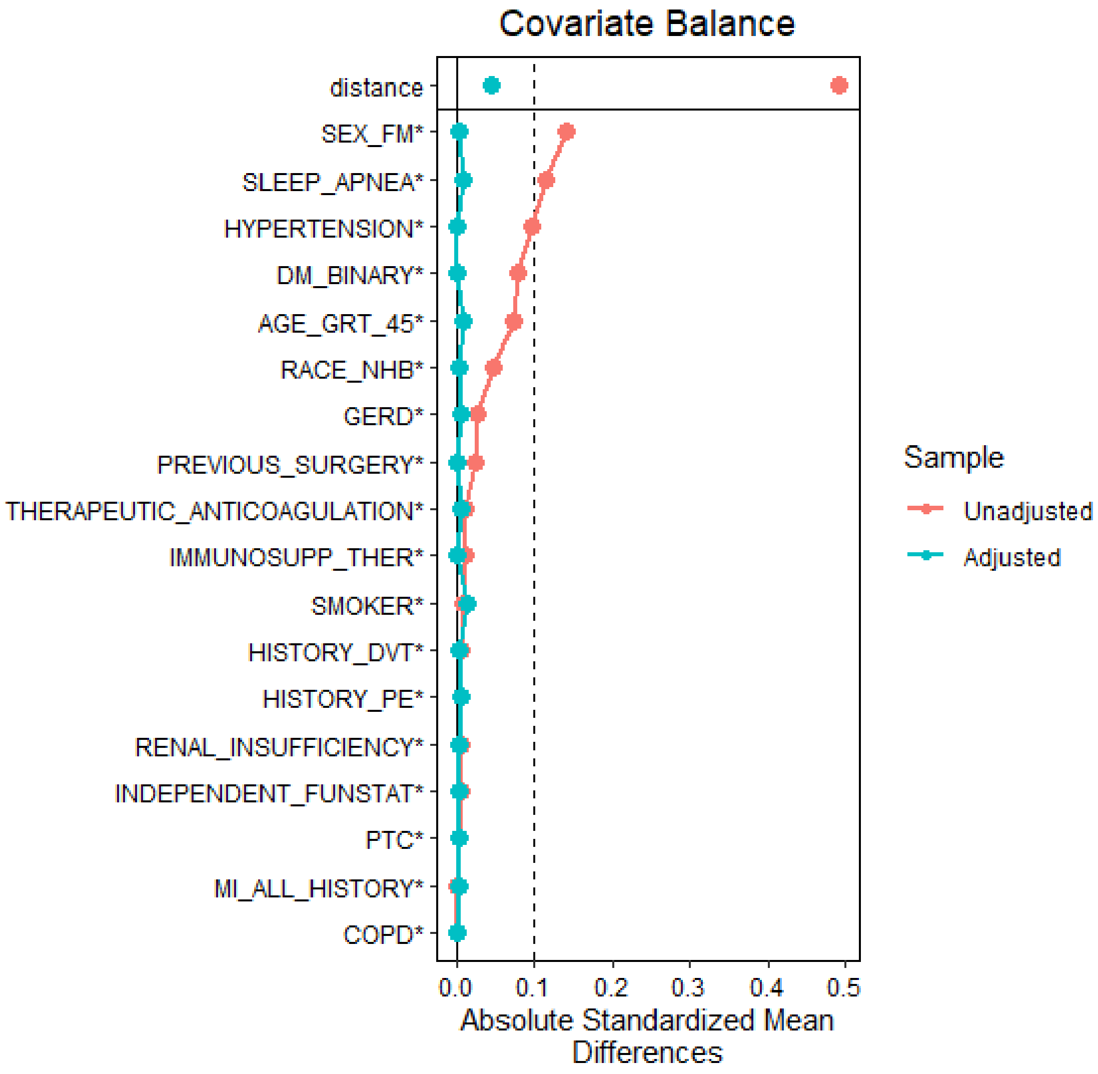

Abbreviations: **SEX_FM:** female sex; **SLEEP_APNEA:** sleep apnea; **HYPERTENSION:** hypertension; **DM_BINARY:** presence of diabetes mellitus; **AGE_GRT_45:** age greater than 45 years old; **RACE_NHB:** non-Hispanic Black race; **GERD:** gastroesophageal reflux disease; **PREVIOUS_SURGERY:** previous abdominal surgery; **THERAPEUTIC_ANTICOAGULATION:** therapeutic anticoagulation use prior to procedure; **IMMUNOSUPP_THER:** chronic immunosuppressant use; **SMOKER:** smoker within one year; **HISTORY_DVT:** history of deep vein thrombosis; **HISTORY_PE:** history of pulmonary embolism; **RENAL_INSUFFICIENCY:** renal insufficiency; **INDEPENDENT_FUNSTAT:** independent functional status; **PTC:** previous percutaneous coronary intervention; **MI_ALL_HISTORY:** history of myocardial infarction; **COPD:** chronic obstructive pulmonary disease.
